# Different Dependence of Tear Strength on Film Orientation of LLDPE Made with Different Co-Monomer

**DOI:** 10.3390/polym11030434

**Published:** 2019-03-06

**Authors:** Yi Ren, Ying Shi, Xuerong Yao, Yujing Tang, Li-Zhi Liu

**Affiliations:** 1 School of Science, Shenyang University of Technology, Shenyang 110027, China; renyi68@aliyun.com; 2 Advanced Manufacturing Institute of Polymer Industry, Shenyang University of Chemical Technology, Shenyang 110027, China; shiying86@aliyun.com; 3 SINOPEC Beijing Research Institute of Chemical Industry, Beijing 100176, China; yaoxr.bjhy@sinopec.com (X.Y.); tangyj.bjhy@sinopec.com (Y.T.)

**Keywords:** LLDPE, tear strength, orientation, film, shrinkage

## Abstract

Crystal orientations, tear strength and shrinkage of Linear Low-Density PolyEthylene (LLDPE) films made with different processes (compressed, cast and blown) were investigated. The films were made with three different LLDPE resins, respectively, which have similar density and molecular weight but are made with different comonomers (1-butene, 1-hexene and 1-octene), in order to investigate if tear strength in Machine Direction (MD) of the LLDPE films made with different comonomer has similar dependence on crystal orientation. Our study indicates that the films made of 1-hexene and 1-octene based LLDPE resins have significantly higher intrinsic tear strength and less decrease in MD tear strength for a given film orientation. That is, for a given orientation in MD, the MD tear drops dramatically for films made with butene-based resin but much less decrease for the films made with hexene and octene-based resins. The shrinkage property at high temperature shows a good correlation with crystal orientation and the fraction of the crystals melted at this temperature.

## 1. Introduction

Linear Low-Density PolyEthylene (LLDPE) is a random copolymer of ethylene and α-olefin made with Ziegler-Natta or metallocene catalysts [1]. LLDPE has a huge market worldwide and is used in many different industries. In fact, a very significant portion of LLDPE resins are used in polymer film industry, mainly for packaging applications. For such applications, mechanical properties, like tear and puncture strength, are very important. It is well known that the films made with LLDPE resins can have better tear and dart strength than the films made with other type of polyethylene, due to higher tie chain concentration introduced by Short Chain Branching (SCB) [2]. For similar branching degree and molecular weight, the tear strength of the film made of LLDPE with longer SCB (hexene and octene comonomers) is significantly better than the films made with butene comonomer [3]. Tie chain concentration is very important to the mechanical property of LLDPE films [4]. Generally speaking, for LLDPE with similar density (or branching degree), the tie chain concentration increases with molecular weight and SCB length determined by comonomer type (1-butene, 1-hexene or 1-octene) [5]. That is, the intrinsic tear strength of compressed films made of LLDPE with different comonomers can be very different [6]. 

From the manufacturing point of view, LLDPE films can be made with different processes, such as, blown and cast film processes, Machine Direction Orientation (MDO), double-bubble and biaxial stretching processes [7]. Studies have shown that the mechanical properties of LLDPE films in different directions can be very different, depending on film orientation [8]. It is well known that the tear strength in Machine Direction (MD) is usually significantly lower than that in Transverse Direction (TD) [9] for LLDPE films. With the increase in crystal orientation, the difference between the tear strength in MD and TD directions becomes more pronounced. In fact, the mechanical failure of the films is closely related to film orientation, such as, cracks are easier to be initiated in MD direction where crystals are preferably aligned [10]. Therefore, for most applications, minimized orientation is desired in order to achieve more balanced tear strength in MD and TD. Reducing crystal orientation is also very helpful to maximize dart and puncture strength.

However, it remains an open question if the dependence of tear strength on crystal orientation for the films made of LLDPE resins with different α-olefin comonomer is same. In fact, many experiments need to be carried out in order to draw the conclusion about the dependence of tear strength on film orientation. In our previous work published in Chinese [11], two of the three film samples studied in the present work were also studied to mainly quantify the differences of tear strength obtained with Elmendorf method at very high deformation speed and with type C (right angle) method at a very slow deformation speed, as the type C method is widely used in China although it is invalid for many film applications. In fact, an in-depth tensile study of polyethylene films at very slow and very fast speeds was also carried out by our group [12]. The different crazing behavior and structural transitions at lamellar level were observed during slow and very fast stretching processes. In the present work, three LLDPE resins with similar density (or branching degree) and molecular weight but different comonomer type (1-butene, 1-hexene and 1-octene, respectively) were used to make films with different processes (compressed, cast and blown). The very different processes can ensure the orientation difference for the films made with the same resin is large enough. The films were investigated for tear strength and crystal orientation. Different dependence of MD tear strength on crystal orientation in MD is observed for the films made with LLDPE resins synthesized with different comonomers. 

## 2. Materials and Methods

### 2.1. Materials

LLDPE resins selected in this study are octene-based 2045 G from Dow Chemical (DOW, Midland, TX, United States), hexene-based 9030 (SINOPEC, Tianjin, China) and butene-based 9085 from Sinopec (SINOPEC, Tianjin, China). All materials were made with Ziegler-Natta catalyst. The resins have very close density; the molecular weight and molecular weight distribution of the resins are also very close, as shown in Table 1. The mole faction of the comonomer for each resin is also listed in Table 1. 

### 2.2. Film Preparation

Blown films were made with COLLIN-BL180-400 (COLLIN, Maitenbeth, Germany) blown film machine with a processing temperature at 195 °C. The screw speed was 30 revolutions per minute (RPM) and the diameter for the die was 80 mm. The blow-up ratio (BUR) was 1.27, which is defined as the ratio between the diameter of the final bubble and the die diameter. The take up ratio (TUR) is 22.5, which is defined as the ratio of the speed at the take-up roll to the speed of the feed at the die exit. The frost line height (FLH) was 85, 63 and 95 mm for 2045G, 9030 and 9085, respectively, with a cooling temperature of 20 °C for dual-lip air ring. The resultant film thicknesses were 30, 28, 28 μm for 2045G, 9030 and 9085, respectively.

Cast films were prepared by LabTec-LCR400 (LabTec, Praksa, Thailand) multilayer co-extrusion cast film machine. The barrel and die temperature was 230 °C and 235 °C, respectively. The temperature of cooling roller was 30 °C. The thicknesses of films were 56, 66 and 60 μm for 2045G, 9030 and 9085, respectively. 

Compressed films were prepared by LabTec-LPS50 (LabTec, Praksa, Thailand). The pre-weighed pellets were evenly placed in a mold and melted at 180 °C for 5 min and then compressed under 5 MPa for 10 min. The film was then cooled to room temperature at a rate of 15 °C/min. The film thickness obtained with this process is around 150 μm. 

### 2.3. Differential Scanning Calorimetry (DSC)

Thermal behaviors of all the resins and the films were studied by Q100 system (TA, New Castle, DE, USA). The tested sample (5mg) was sealed in an aluminum holder and was heated up from 25 °C to 200 °C at a rate of 10 °C/min with the protection of nitrogen gas. The melting behavior of the films can be studied with the first heating of the films. The resin samples were kept at 200 °C for 5 min and were then cooled down to 25 °C at 10 °C/min to investigate the non-isothermal crystallization of these resins. The resin samples were subsequently heated back to 200 °C at the same rate to investigate their melting behaviors. 

### 2.4. Small Angle X-ray Scattering (SAXS)

The data were collected with NANOSTAR U SAXS instrument (Bruker, Karlsruche, Germany) operated at 40 kV and 650 μA. The data collection time for each sample was 180 min. The wavelength (λ) used was 0.154 nm (Cu Kα) and pinhole collimation system was employed to tune the X-ray beam. A Hi-Star area detector (Bruker, Madison, WI, United States) with 1024 × 1024 pixels and each pixel being 100 μm was used for data collection. The distance between the sample and the detector was 1070 mm, with a covered q-rang from 0.09 to 2.0 nm^−1^, where *q* represents the scattering vector (*q* = 4πsinθ/λ and 2θ represents the scattering angle). The collected data were corrected for air background before any analysis. Evaluation of Herman’s orientation of the films was also carried out e based on the SAXS data with MD as the reference direction. 

### 2.5. Elmendorf Tear Strength

The Elmendorf tear strengths of the films were measured by PROTEAR (Thwing-Albert, West Berlin, NJ, USA) according to the standard of ASTM-D-1922. Film specimens were cut to form a rectangle 76 mm in width by 63 mm in length (tearing direction). Two sets of specimens were cut from each film sample so that their sides are parallel to MD and TD directions, respectively, for the corresponding MD and TD tear strength test. Ten specimens were prepared and tested for each measurement in order to get a good average on tear strength. The film specimen is clamped between two clamps and pre-notched by a razor blade at the bottom between the clamps. The uncut portion of the film is 43 mm in notching direction. The tear measurement is done by releasing the pendulum aligned horizontally to tear the un- notched portion of the film. The loss energy due to tearing the specimen is used to calculate the Elmendorf tear resistance.

## 3. Results and Discussion

Although the LLDPE resins studied in this work have similar density and molecular weight, the crystallization behavior can still have appreciable differences, as LLDPE is basically a mixture of the molecules with different polyethylene sequence distribution which can be different for these resins, besides the difference in co-monomer type. These differences can lead to different crystallization behavior during a same cooling process and different subsequent melting behavior. The differences in crystallization dynamics and the corresponding melting behavior of the resins can be studied with DSC and the learning would be very helpful for understanding different film-making processes used in this work. 

### 3.1. Non-Isothermal Crystallization of the LLDPE Resins and Thermal Property of the LLDPE Films Made of the Resins With Different Processes

**Figure 1:**
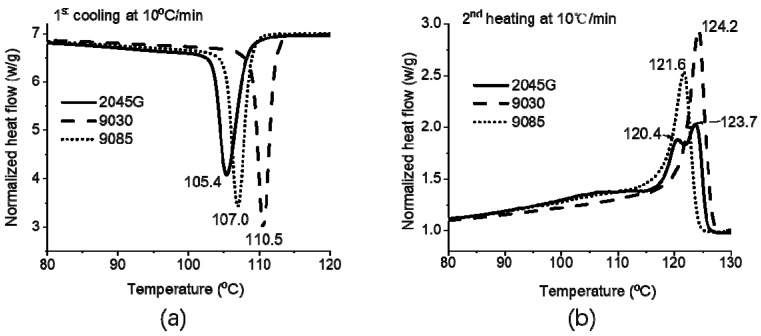
DSC cooling (−10 °C/min) thermograms (**a**) of the resins studied and the subsequent melting thermograms (**b**).

The non-isothermal crystallization during a cooling at 10 °C/min from melt and a subsequent heating at 10 °C/min were studied with DSC for the three LLDPE resins made with different comonomers. The cooling and subsequent heating traces are shown in Figure 1a,b. As shown in this figure, the crystallization temperature during the cooling process for 2045G, 9085 and 9030 sample is 105.4, 107.0 and 110.5 °C, respectively, indicating that 9030 with a hexene co-monomer has a stronger crystalline ability than 9085 and 2045G samples. The 2045 resin with octene comonomer has the weakest crystalline ability. The resins were studied as received and without further treatment; the difference seen from the DSC study can be attributed to the difference in PE sequence distribution or possible additive effect or both of the effects. In fact, the melting behavior typically cannot be affected that much by possible additives and can provide useful information to differentiate the three resins. Figure 1b does show that 9030 has a significant higher melting point than the other two materials, suggesting this resin is very likely to have more high-density fraction. On the other hand, the heating thermogram of 9030 resin also shows less melting in the temperature range from 90 to 110 °C than the other two materials, suggesting that fewer small crystals were formed during the cooling process. It is also noticed that double melting peaks (120.4 and 123.7 °C) are observed for 2045G resin as seen in Figure 1b. The melting behavior of 9085 is similar to 2045 G but with a lower melting point (121.6 °C). The results suggest that 2045G is likely to have a bi-model distribution in terms of the short chain branching of the molecules, while the other two materials are not. The crystallinity of 2045G, 9085 and 9030 is 42.4, 39.7 and 37.8%, respectively. 

**Figure 2:**
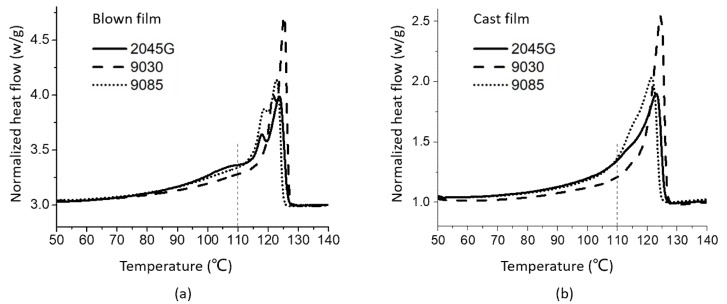
DSC heating thermograms of LLDPE films made with blown film process (**a**) and cast film process (**b**).

**Figure 3:**
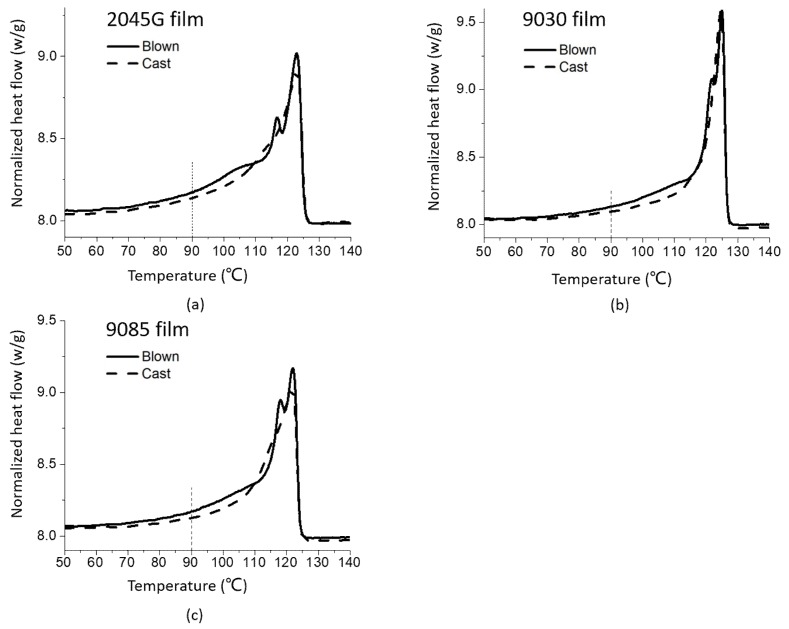
DSC heating thermograms of the cast and blown films made with different LLDPE resins (**a**) 2045G, (**b**) 9030 and (**c**) 9085.

LLDPE films made by blown and cast film processes were also studied with DSC technique. As shown in Figure 2, the melting behavior of any of the films is quite different from its corresponding melting thermogram obtained from the same resin but underwent the non-isothermal crystallization during the cooling at 10 °C/min (Figure 1b). Double melting peaks are observed for all three blown films (one peak and a shoulder for 9030). For the cast film process, only one melting peak is observed for the film made with 9030, but one melting peak and one shoulder are present for the films made of 2045G and 9085 resins. For a cast film process, the extruded film immediately reaches a cooling roll, so crystallization finishes very fast. While for our blown film process, the frost line high was from 60 to 100 mm, suggesting that the crystallization finished at a slow process. This may explain why double melting peaks above 110 °C (Figure 2) are observed for the blown films. The slow crystallization process allowed the component with a strong crystalline ability to crystallize first and the component with a weak crystalline ability to crystallize later to form smaller crystals represented by the lower melting peak of the observed double peaks. As can be seen from the dash line at 110 °C, the average melting point for the blown films is higher (further away from the line) than the average melting point for the cast films (close to the line). The direct comparison of the melting thermograms for the cast and blown films made of the same resin are shown in Figure 3. It can be seen clearly from this figure that more crystals with even lower melting point are formed during blown film process than cast film process, as can be seen in the temperature range right above 90 °C (the dash line). The crystals with lower melting point could affect the shrinkage property of the films at elevated temperature, which will be addressed in later section.

### 3.2. Structure and Orientation of LLDPE Films Made with Different Resins and Different Processes 

**Figure 4:**
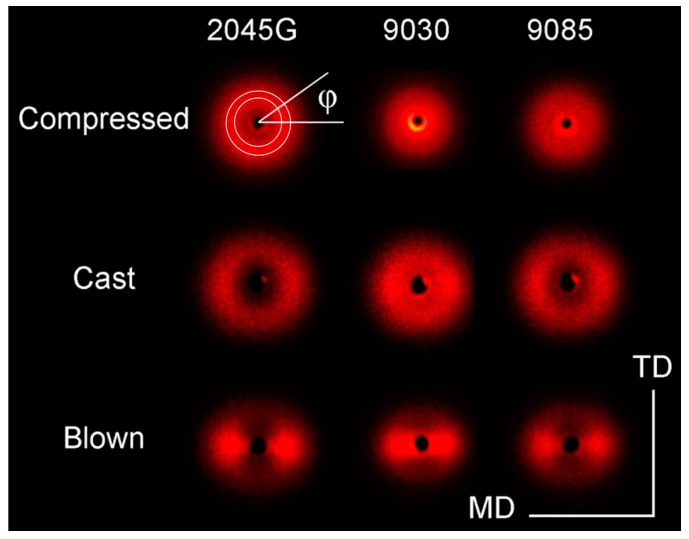
SAXS images of LLDPE films made by the three different processes with different resins.

The SAXS patterns of the LLDPE films processed by the three different processes for each resin are shown in Figure 4. As expected, no crystal orientation is seen for the compressed films, while weak crystal orientation is observed for the cast film, suggested in Figure 4 by more scattering in MD than in TD direction. During the cast and blown film processes, some oriented lamellar stacks with their normal direction being approximately along MD direction are formed, which leads to more SAXS in MD direction than in TD direction. In fact, most anisotropic SAXS scattering patterns (two-dots scattering patterns) are observed in case of the blown films (Figure 4), indicating that stronger crystal orientation is formed for the blown films. 

**Figure 5:**
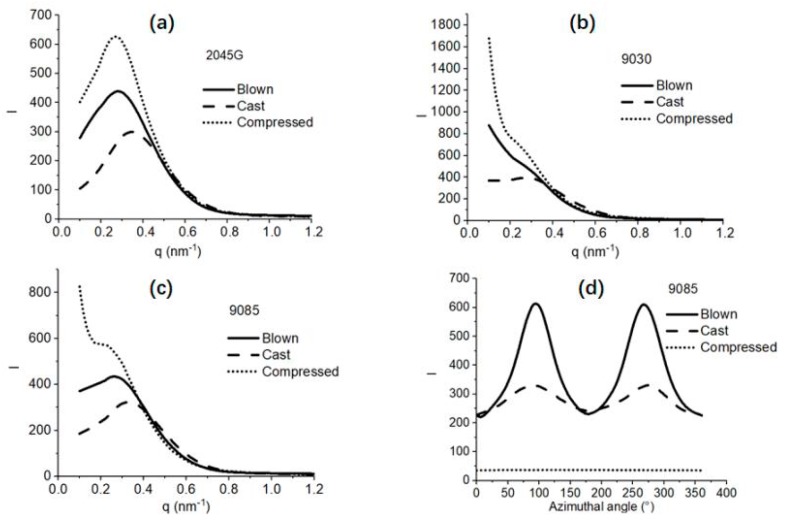
The average SAXS profiles (over 360°) obtained from the SAXS images shown in Figure 4 for the films made with (**a**) 2045G, (**b**) 9030 and (**c**) 9085. As an example, the azimuthal SAXS scattering profiles crossing the scattering maxima for LLDPE 9085 film are shown in figure (**d**), which is used for evaluation of Herman crystal orientation function with MD direction as a reference direction.

**Figure 6:**
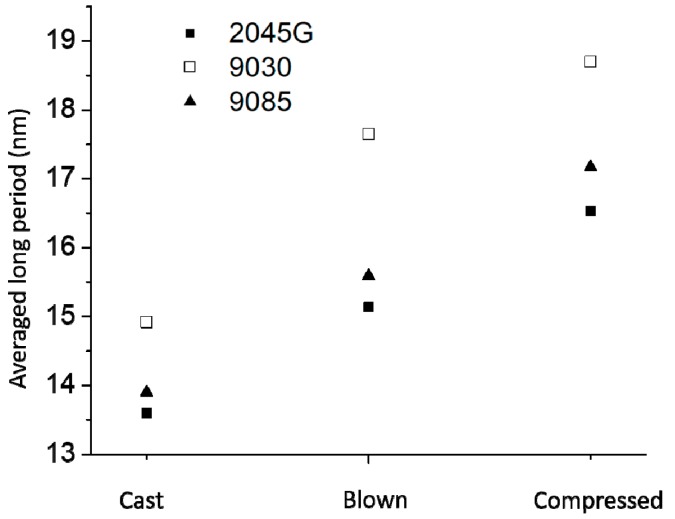
The long period of cast, blown and compressed films made with 2045G, 9030 and 9085 resins.

The information on lamellar packing can also be obtained from the scattering images. The average scattering profiles over 360° are shown in Figure 5. The scattering profiles show that a well-defined scattering peak is obtained for the films made with 2045G, indicating that a well-organized lamellar packing structure is formed in these films. Less organized lamellar packing structure is formed in the films made of 9085 resin by showing less well–defined scattering peaks and least organized lamellar packing is formed in the films made with 9030 resin, as no well-defined scattering peak is present. Another important structural parameter on the lamellar stacks is the average long period, meaning the average total thickness of crystalline lamellae and amorphous lamellae or the average interdistance of neighboring crystalline lamellae. The actual long period can be obtained with a few different approaches [13,14]. In the present work, Lorentz-correction was applied to the scattering curves (not shown) to evaluate the long period, as scattering maxima is absent for some of the scattering profiles. The long periods for the 9 films made with different resins and by different processes are shown in Figure 6. It can be seen from this figure that the long period of the film made with the same resin can vary a lot with different film process. The long period for the compressed film is the largest, while it is the smallest for the cast film. This trend is understandable considering the speed of the cooling for the different processes. Generally speaking, thicker crystalline lamellae (higher melting point) and larger long period is formed for a slower crystallization process and vice versus. For the compressed films, the films were cooled down gradually inside an oven, thus it was supposed to be much slower than the other two processes. In the previous thermal section, it was addressed that the cooling for the blown film process was supposed to be slower than the cast film process. In fact, this is confirmed by the SAXS study based on the larger long period of the blown film than the cast film made of the same resin. 

It is also seen from Figure 6 that for the same film process, the long period of the film made with 2045G is the smallest, while the long period is the largest for the film made with 9030 resin. Although the long period of film made with butene-based 9085 is very close to the film made with octene-based 2045G for the same process (Figure 6), there is a huge difference in intrinsic tear strength for these two films (will be discuss in later section), implying that the films, made with very similar resins in terms of density (crystallinity) and molecular weight and with similar structural parameter like long period, can have very different tear strength. In fact, tie-chain concentration (not reflected by typical structures) or the connected degree among the crystals at chain level is a very important governing factor for intrinsic tear strength of polyethylene film.

The mechanical properties of polyethylene films closely relate to crystal orientation which can be evaluated with the 2D SAXS. The crystal orientation can be quantitatively evaluated with azimuthal intensity profile crossing the scattering maxima within a selected q-range as shown in Figure 4 for the 2D scattering pattern obtained from compressed 2045G film. The azimuthal SAXS scattering profiles crossing the scattering maxima are shown in Figure 5d for LLDPE 9085 film for example. These azimuthal scattering profiles are used for evaluation of Herman orientation function with MD direction as a reference direction.

Herman’s orientation function [15], f, is defined as follows:

(1) $$f=\frac{1}{2}\left(3\langle \cos^{2}\phi \rangle -1\right)$$

(2) $$\langle \cos^{2}\phi \rangle =\frac{\int_{0}^{\frac{\pi }{2}}I\left(\phi \right)\sin\phi \cos^{2}\phi d\phi }{\int_{0}^{\frac{\pi }{2}}I\left(\phi \right)\sin\phi d\phi }$$

where ϕ is azimuthal angle shown in Figure 4. For perfect orientation with a reference direction in orientation direction, *f* equals to 1, while *f* equals to −1/2 for a perfect orientation with a reference direction 90 degree off the orientation direction. For a random system without preferred orientation, *f* equals to zero. For our data processing, MD was used as a reference direction; the selected q range for generating azimuthal profiles for orientation evaluation was 30 pixels. The calculated Herman’s orientation values for the cast and blown films made with each of the resins are listed in Table 2. It can be seen from this table that the film made with blown film process has significantly larger orientation than the corresponding cast film. 

### 3.3. Intrinsic Tear Strength and MD Tear Strength for Oriented Films

As discussed above, films made by industry processes (cast or blown) are oriented. The mechanical properties of the films are very much dependent on film orientation. The intrinsic tear strength for films without any orientation is a better way to compare the tear strength for the films made with different LLDPE resins. In the present work, compressed films were made for Elmendorf tear test; the normalized intrinsic tear strength values for the compressed films made with the three LLDPE resins are listed in Table 3, together with the MD and TD tear strength values of the cast and blown films made with the three LLDPE resins. 

**Figure 7:**
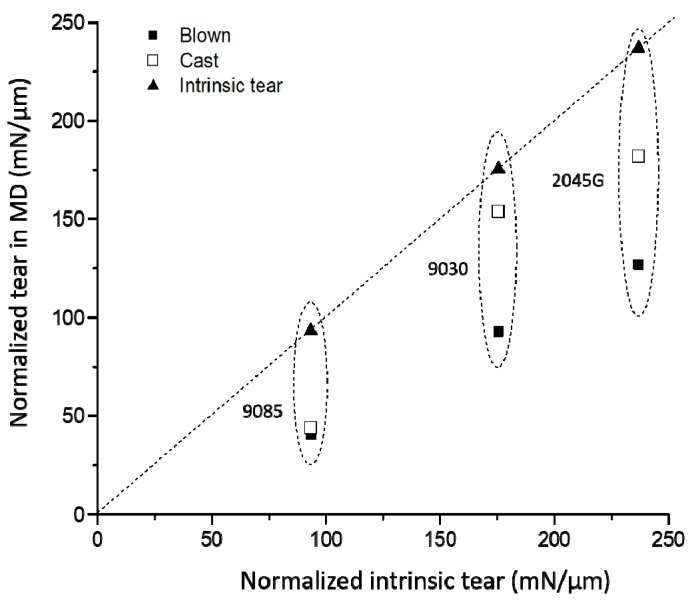
Intrinsic tear strength vs. tear strength in MD for the films made with the three LLDPE resins.

For most film applications, balanced MD and TD tear strengths are desired, as the mechanical failure of a film is usually determined by the weak strength direction, such as, puncture and tear. However, practically, it is not easy to achieve the balanced mechanical properties, as crystals in the film made with typical film making processes have a preferred orientation direction. It is well known that once a film is oriented, the tear strength in the oriented direction drops significantly. A film gets damaged easier in the direction where crystals are oriented, leading to lower tear strength in the orientation direction. This trend can also be seen from Table 3 for the films studied in this work. The tear strength in MD direction can be significantly smaller than its intrinsic tear strength, due to the preferred orientation in MD, while TD tear strength can be much larger than its intrinsic tear strength. As MD tear is more important for a film performance, the MD tear strength is plotted against intrinsic tear strength in Figure 7. It can be seen from Table 3 and Figure 7 that the intrinsic tear strength increases significantly with increasing the short chain length determined by the co-monomer type. The intrinsic tear strengths of the films made with LLDPE with octene and hexene co-monomers are remarkably better than the film made with LLDPE with butene co-monomer. More importantly, the MD tear strength of cast and blown films made with butene co-monomer drops dramatically relative to the corresponding intrinsic tear strength but the tear strengths in MD, relative to the corresponding intrinsic tear strength, holds better for the cast and blown films made with hexene and octene co-monomers. 

### 3.4. MD Tear Strength Dependence on Crystal Orientation and Co-monomer Type of LLDPE

**Figure 8:**
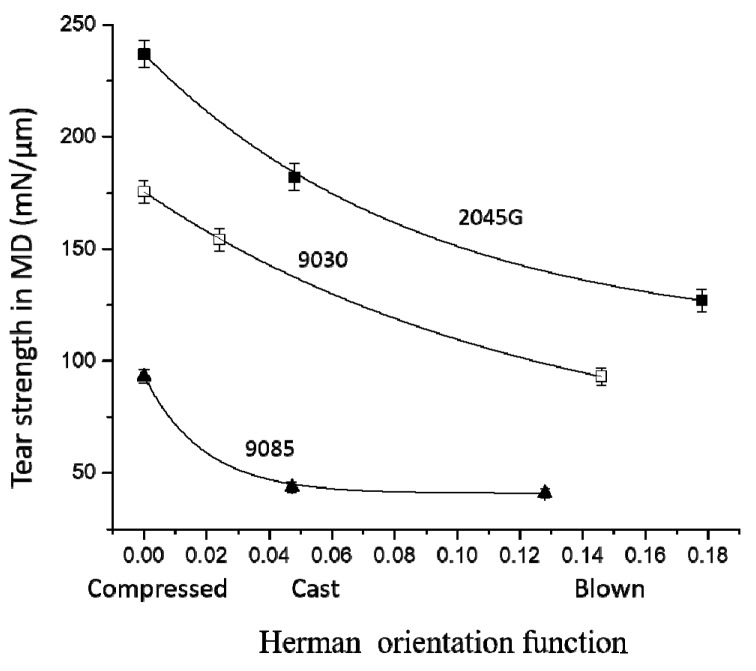
MD tear strength vs. crystal orientation for the films made with the three resins.

**Figure 9:**
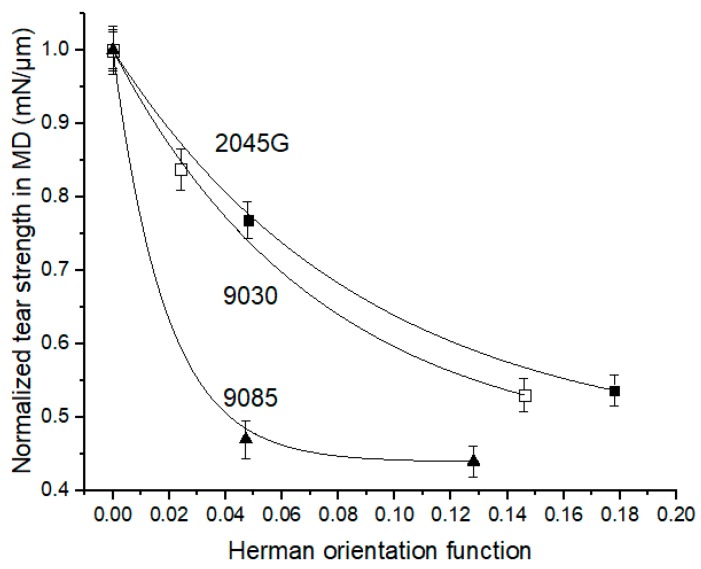
Normalized MD tear strength (by its corresponding intrinsic tear strength) as a function of crystal orientation.

Usually, the more oriented a film is, the more loss its tear strength in MD direction bears. As cast and blown films made with different resins usually have different orientation degree even under the same processing condition, it is difficult to evaluate the difference in the dependence of MD tear strength on crystal orientation for the films made by different LLDPE resins. In the present work, three films with different orientation degree were made for each of the three resins with butene, hexene and octene co-monomer, respectively, which makes such an evaluation possible. As shown in Figure 8, the MD tear strength decreases quickly with increase in crystal orientation for the films made with any of the resins. It seems that the MD tear strength decreases faster with increase in crystal orientation for the films made with LLDPE with butene comonomer. The different response to orientation can be better viewed in Figure 9 from the plot of normalized MD tear strength (by its the intrinsic tear strength) versus crystal orientation. It can be seen from Figure 9 that at a given orientation degree, the film made with a longer short chain branching always has a larger MD tear strength. The length of short chain branching plays a key role in the dependence of MD tear strength on crystal orientation. Figure 9 clearly shows that as increase in crystal orientation the MD tear strength of the films made with butene based LLDPE drops much faster than films made with the hexene and octene based LLDPE. As increase in crystal orientation, the MD tear strength for the films made with hexene based LLDPE decreases slightly faster than the film made with the octene based LLDPE. As shown in Figure 9, for Herman orientation value of 0.05, the MD tear strength still remains 77% of its intrinsic tear strength for the film made with 2045G with octene co-monomer, while it drops to 47% of its intrinsic tear strength for the film made with 9085 with butene co-monomer. In another word, the MD tear strength for the films made with 2045G and 9030 with hexene and octene co-monomers are much less sensitive to orientation than the film made with 9085 with butene co-monomer. It should also be pointed out that the compressed films with significantly larger thickness than films made with other processes were used for evaluation of intrinsic tear strength. As the cast films have very weak orientation, especially for the two films made with 2045G and 9030, the averaged tear strength in MD and TD direction can be used to approximately compare with its corresponding intrinsic tear strength obtained from the compressed films. It can be seen from Table 3 that the average from 9030 cast film is very similar to the obtained intrinsic tear strength and the average from 2045G is about 20% smaller than the obtained intrinsic tear strength. The larger difference for 2045G does not change the above conclusion on tear dependence on film orientation but make it more solid. In fact, smaller intrinsic tear strength for 2045G leads to the conclusion that the tear strength in MD direction for the film made with octene based co-monomer (2045G) is even less sensitive to crystal orientation than described above. 

### 3.5. Shrinkage Property at High Temperature vs. Crystal Orientation and Melting Behavior

**Figure 10:**
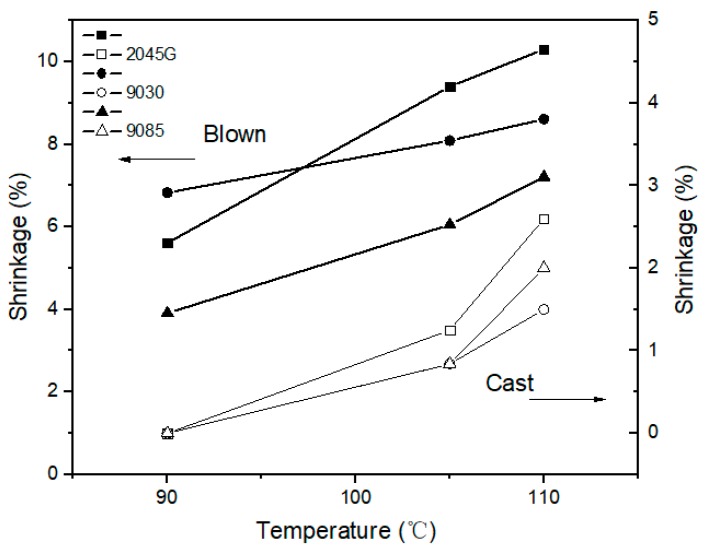
The shrinkage at different temperatures for the cast and blown films made with the three LLDPE resins.

The film shrinkage values at different temperatures are shown in Figure 10. It can be seen from this figure that the shrinkage measured from the blown films is obviously larger than the cast films for all three series of samples. The blown films start to shrinkage at 90 °C, while the cast films do not shrink at this temperature. This can be interpreted based on the thermal data shown in early section. As shown in Figure 3 that more crystals with lower melting point are formed during blown film process than cast film process, as indicated by the dash line at 90 °C in that figure. This is why the blown films shrink at 90 °C, while the cast films hold pretty well. In fact, the average melting point for the blown films is higher than the average melting point for the cast films, as indicated by the fact that the overall melting for the blown film is within a higher temperature range than the cast films (Figure 3). The blown film made with octene based 2045G also has more crystals with significantly lower melting point (below 110 °C, see Figure 3) than the other two blown films, leading to largest shrinkage at 110 °C for 2045G blown film. 

**Figure 11:**
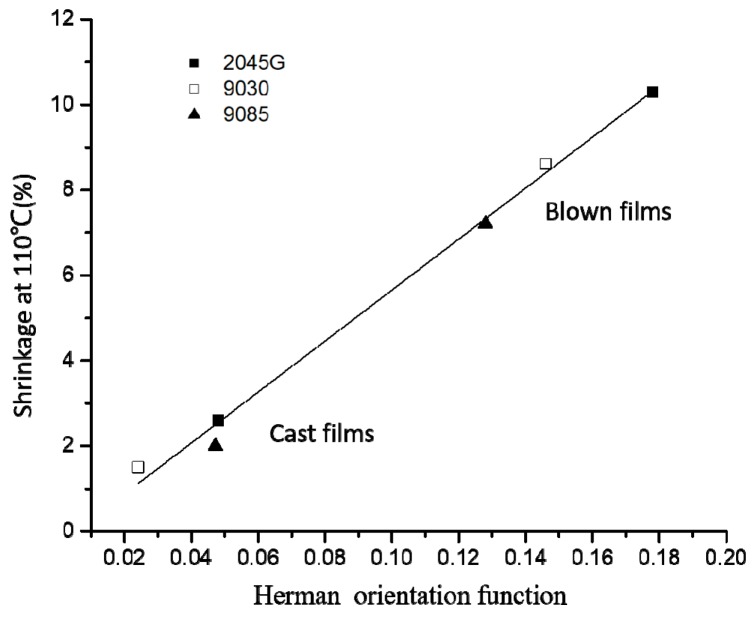
The shrinkage at 110 °C vs. crystal orientation for all cast and blown films made with three different resins.

On one hand, crystal orientation plays a big role for the shrinkage property. On the other hand, the shrinkage property at a given temperature can be also affected by the crystal fraction with low melting point. The shrinkage at 110 °C for all cast and blown films made with the three different resins is plotted in Figure 11 as a function of crystal orientation. It is seen from this figure that although the data is from both cast and blown films made with three different LLDPE resins, the shrinkage correlates linearly with crystal orientation.

## 4. Conclusions

In the present work, the films made with very similar resins in terms of density (crystallinity) and molecular weight but different co-monomer type (butene, hexene and octene, respectively) were studied with different techniques. The long period of film made with butene-based 9085 is very close to the film made with octene-based 2045G but there is a huge difference in intrinsic tear strength for these two films due to different co-monomer type. That is, very similar lamellar packing structure (crystallinity/density and long period) does not necessarily make similar tear strength of the LLDPE films., The tie-chain concentration (not reflected by typical structures) or the connected degree of the crystals at chain level is an important governing factor for intrinsic tear strength of polyethylene films.

MD tear strength for the films made with LLDPE with butene co-monomer decreases the fastest as increase in crystal orientation, such as, for a given Herman orientation value at 0.05, the MD tear strength for the film made with octene co-monomer can still remain 77% of its intrinsic tear strength, while it drops to 47% of its intrinsic tear strength for the film made LLDPE with butene co-monomer (Table 4). As increase in crystal orientation, the MD tear strength for the films made with hexene based LLDPE decreases slightly faster than the film made with the octene based LLDPE at a given orientation value.

For shrinkage property at elevated temperatures, on one hand, crystal orientation degree plays a big role. The shrinkage increases linearly with increase in crystal orientation, confirmed by the results from the cast and blown films made with LLDPE resins with different co-monomers (butene, hexene and octene, respectively). On the other hand, at a certain elevated temperature, the shrinkage property can also be affected by the crystal fraction with low melting point. In fact, our study shows that more crystals with lower melting points (say around 90°C) are formed during blown film process than cast film process, especially for films made with bi-model LLDPE resin (2045G). 

## Figures and Tables

**Table 1:** The characteristics of the resins used.

Sample	Comonomer Type	Density (g/cm^3^)	MWD	MW	Comonomer Content (mol%)
2045G	octene	0.920	3.85	1.27 × 10^5^	2.4
9030	hexene	0.919	4.74	1.10 × 10^5^	3.8
9085	butene	0.920	4.21	1.33 × 10^5^	3.9

**Table 2:** Herman’s Orientations function for all the films study.

Sample Designation	Compressed	Cast	Blown
2045G	0	0.048	0.178
9030	0	0.024	0.146
9085	0	0.047	0.1328

**Table 3:** The intrinsic Elmendorf tear strength and MD and TD Elmendorf tear strength of the films made with the three LLDPE resins.

Sample Designation	Tear Strength (mN/μm)				Intrinsic Tear (mN/μm)
	Blown		Cast		
	MD	TD	MD	TD	
2045G	127	411	182	198	237
9030	93	287	154	191	176
9085	41	219	44	105	93

**Table 4:** Summary of the key results obtained from the films made with the three resins.

Sample		2045G	9030	9085
Comonomer type		Octene	Hexene	Butene
Density (g/cm^3^)		0.920	0.919	0.920
MW(×10^5^)		1.27	1.10	1.33
Long period (nm)				
	Cast	15.1	17.7	15.6
	Blown	13.6	14.9	13.9
Intrinsic tear (mN/μm)		237	176	93
MD tear /intrinsic tear at the orientation of 0.05		77%	75%	47%
